# An Alternative Prediction Equation for Evaluation of Six-Minute Walk Distance in Stable Coronary Artery Disease Patients

**DOI:** 10.3389/fphys.2022.844847

**Published:** 2022-03-31

**Authors:** Helena Lenasi, Ana Novak, Borut Jug, Edvin Dervišević, Damir Karpljuk, Mateja Videmšek, Maroje Sorić, Vedran Hadžić

**Affiliations:** ^1^ Institute of Physiology, Faculty of Medicine, University of Ljubljana, Ljubljana, Slovenia; ^2^ Sports & Medicine Department, Faculty of Sport, University of Ljubljana, Ljubljana, Slovenia; ^3^ Department of Vascular Diseases, Division of Internal Medicine, University Medical Center, Ljubljana, Slovenia; ^4^ Department of Sport and Exercise Medicine, Faculty of Kinesiology, University of Zagreb, Zagreb, Croatia

**Keywords:** cardiac rehabilitation (CR), coronary artery disease, cardiorespiratory fitness (CRF), six-minute walk test (6MWT), six-minute walk distance (6MWD), NYHA class, waist circumference (WC)

## Abstract

**Background:** As cardio-vascular diseases are the leading cause of death worldwide, establishing measures to improve cardiovascular health is of crucial importance. Exercise plays an essential role in cardiac rehabilitation of patients with coronary artery disease (CAD), in whom an evaluation of the cardiorespiratory fitness (CRF) is necessary. CRF of CAD patients could be assessed using 6-min walk test (6MWT), and the results interpreted by using Enright-Sherill prediction equation which has mainly been designed and evaluated for a healthy population. Hypothesizing that the Enright-Sherill prediction equation might not be best suited for CAD patients, our aim was to reevaluate this equation in CAD patients, and potentially establish a more accurate 6MWD prediction equation to be applied in these patients.

**Methods:** 6MWD was measured in a cross-sectional study in 67 CAD patients (44 women) who were members of the Coronary club Ljubljana, Slovenia. In addition, the predicted 6MWD was calculated for men and women using Enright-Sherill gender specific regression equation. Multivariate regression analysis was used to obtain a new prediction equation, and the agreement between the measured and the predicted 6MWD analyzed using the repeated measures ANOVA.

**Results:** Men achieved 451 ± 122 m and women 485 ± 69 m without significant differences between sexes (F = 0.022, *p* = 0.882) when adjusted for age, height, body mass, and waist circumference. When comparing the measured (473 ± 91 m) and the predicted (422 ± 57 m) values of 6MWD in CAD patients we found that the Enright-Sherill prediction equation significantly (F = 27.734, *p* < 0.001) underestimated the 6MWD by 52 ± 81 m. A significant regression equation was established [F (3,63) = 44.663, *p* < 0.001], with a *R* ^2^ of 0.680 where 6MWD equals 1,057 m—4.966 x age (years)—0.614 x WC (cm)–68.629 x NYHA class.

**Conclusion:** The results of this study stress the importance of regular and actual walking ability testing in patients with stable CAD to obtain their CRF, rather than simply predicting it from regression equations obtained from non-representative or non-comparable samples. Our developed prediction equation warrants additional validation and may represent a good substitute for currently used predictions obtained from a healthy population.

## Introduction

Six-min walk test (6MWT) is a simple field based functional test used to evaluate walking ability in healthy individuals (Casanova et al., 2011) and in patients with various diseases and of different age (Bohannon and Crouch, 2017). The main outcome of the test conducted indoor on a 30-m-long corridor with two turning points is 6-min walk distance (6MWT) (AmericanThoracicSociety, 2002). 6MWT is commonly used in outpatient cardiac rehabilitation programs as a follow-up tool to assess cardio-respiratory fitness (CRF) in patients with coronary artery disease (CAD) and congestive heart failure (Bellet et al., 2012). Studies have reported a good correlation between the 6MWD and clinical status of patients following cardiac rehabilitation, with a minimal clinically important difference of 25 m (Gremeaux et al., 2011), and clinically acceptable reproducibility (Gayda et al., 2004) in CAD patients. It has been suggested that 6MWD can predict future cardiovascular events in patients with stable CAD (Beatty et al., 2012). Moreover, the calculation of gait speed from 6MWT was suggested as a simple risk stratification tool in older CAD patients (Kamiya et al., 2017).

Life-long outpatient cardiac rehabilitation of CAD patients in Slovenia has a 35-year-long tradition of patient organizations (i.e., coronary clubs), which provide supervised exercise training, psychosocial support, access to verified health-related information and continual control of risk factors. The country-wide network encompasses 17 regional coronary clubs located in 81 towns throughout the country, including 153 exercise groups with 3.818 active members (Hadzic et al., 2017). Within those clubs, the 6MWT is performed annually to evaluate CRF of CAD patients. The real achieved 6MWD of a patient is usually compared with the estimated 6MWD, that is calculated from a prediction equation, and the data are presented as a percentage of the predicted value. This comparison is necessary to interpret the fitness status of an individual patient and to modify his exercise plan accordingly.

A thorough systematic review has identified 17 different prediction equations from a healthy population in which the estimated independent predictors of the 6MWD were (depending on the particular equation used) body height, weight, age, sex, and the heart rate assessed during the test, but it was concluded that there are large differences in the predicted distance among the studies (Singh et al., 2014). However, prediction equations based on the predictors obtained from patients are lacking, and the measured values in CAD patients are usually compared to a prediction acquired from a healthy population. Accordingly, evaluation of the CRF based on prediction of 6MWD in an individual patient might be a source of error, as the predicted 6MWD may overestimate or underestimate the true walking ability of the patient, depending on specific equations used in practice. Therefore, we believe that it is necessary to establish a more accurate and specific equation for specific target groups which could be more reliable to use in clinical practice than the commonly used equations based on the data from a healthy population. This is clinically important, as in clinical practice overestimation of patient’s ability may lead to adverse cardiovascular events with exercise (Goodman et al., 2013). On the other hand, underestimation may also affect exercise prescription in patients, causing them to exercise with intensity and volume below the threshold necessary to achieve any improvements.

The main goal of our study was to reevaluate the accuracy of the Enright-Sherill equation (Enright and Sherrill, 1998) which is currently used for prediction of 6MWD in CAD patients in regional coronary clubs. Moreover, we aimed to establish a new multivariate regression model including the commonly used independent predictors of 6WMD, potentially applicable for further validation.

In our regression model, we aimed at including some additional predictors that have not been tested before and we believe might improve the accuracy of the predicted 6MWD, such as waist circumference (WC) and the New York Heart Association (NYHA) class of the patient. WC and waist to height ratio (WHtR) were both shown to have the best discriminatory power in predicting cardiovascular risk factors compared to other indices such as body mass, body height and body mass index (Correa et al., 2016). Moreover, NYHA classification (I–IV), based on patient’s and physician’s assessment of cardiac symptoms including dyspnea, angina, and fatigue at different levels of physical activity, has been mostly used in clinical practice, and studies have stressed a need for a better understanding of the relationship between NYHA class and 6MWD (Yap et al., 2015). We believe that NYHA should be taken into consideration when assessing CRF in CAD patients, and above all, designing their individual rehabilitation program. Accordingly, we hypothesized that NYHA and potentially anthropometric parameters, such as WC and/or WHtR class might importantly contribute to the prediction of 6WMD in CAD patients.

## Materials and Methods

### Participants

This was a cross-sectional study enrolling 67 CAD patients (44 women, 23 men) from the regional coronary club (Ljubljana, Slovenia). All patients have provided written informed consent to participate during their regular annual CRF testing. The study was approved by the Board of Ethics in Sport at the Faculty of Sport in Ljubljana (number 9/2020-491), and accordant to the principles outlined in the Declaration of Helsinki.

Most of the patients had suffered an ischemic heart attack (52%), 24% had percutaneous coronary intervention, and 15% coronary artery bypass grafting. Most common comorbidities were arterial hypertension (65%) and hyperlipidemia (45%). Patients were classified into different NYHA classes by their treating cardiologist based on clinical appraisal and history of exertion-related symptom onset (I–no limitation of physical activity; II–slight limitation with ordinary physical activity yielding fatigue, palpitation, dyspnea or other cardiovascular symptoms; III–marked limitation; IV–unable to carry out any physical activity without symptoms). There were 11 (16%), 35 (52%) and 21 (31%) patients in NYHA I, NYHA II and NYHA III class, respectively. Patients from NYHA IV class did not participate in the study.

### Measurements

All measurements including the 6MWT were performed at the gym of the regional coronary club. Prior to 6MWT, we have measured body height and body mass using a stadiometer and a medical scale (models 222 and 762, respectively; Seca Instruments Ltd., Hamburg, Germany). WC was measured using a tape-meter and according to the WHO STEPwise Approach to Surveillance protocol (STEPS) at the midpoint between the lower border of the rib cage and the iliac crest (Albu et al., 2010).

6MWT was conducted according to the guidelines of the American Thoracic Society (AmericanThoracicSociety, 2002). In general, the test was performed in a 30-m-long corridor free of obstacles, with two turning points and marks placed at a 3-m distance from each other (AmericanThoracicSociety, 2002). Patients were instructed to walk as far as possible for 6 min around the given course, covering as much ground as possible during that time. There was no warm-up prior to the test, and all participants were resting for 10 minutes before starting the test. We have calculated the predicted 6MWD from Enright-Sherill sex specific regressions equations (Enright and Sherrill, 1998) for men and women: for men, 6MWD = (7.57 x height cm)–(5.02 x age)–(1.76 x weight kg)–309 m, and for women, 6MWD = (2.11 x height cm)–(2.29 x weight kg)–(5.78 x age) + 667 m. 6MWD = (0.88 x height cm)–(2.11 x weight kg)–(5.44 x age) + 896 m.

### Statistical Analysis

All data were analyzed using the IBM SPSS Software for Windows (version 25, SPSS Inc., Chicago, Illinois, United States). Categorical variables are displayed as numbers and percentages, while continuous variables as means and standard deviations. All numeric variables were firstly checked for normality of distribution with Shapiro-Wilk`s test.

The agreement between the measured and the predicted 6-min walk test distance was first analyzed using Bland–Altman analysis (Ranganathan et al., 2017). Repeated measures ANOVA was used to analyze differences between measured and predicted 6MWD. The differences in 6MWD between different groups (e.g., men vs. women, different NYHA classes, etc) were assessed using univariate analysis of variance adjusted for specified covariates (age, height, body mass and WC). Bonferroni correction for multiple comparisons was used when appropriate. The reported effect size from univariate model was partial eta squared. We have performed repeated linear regression calculation first adding individual predictors and then combining significant ones into the final multiple linear regression model. We have examined potential collinearity, and we present only models with variance inflation factor below 2. A significance level of 0.05 was used for all tests. An *a priori* sample size calculation for multiple linear regression model (fixed model, *R* ^2^ deviation from zero) was conducted using G*Power3 (Faul et al., 2009) using a large effect size (d = 0.35), an alpha of 0.05 and three predictors. The result showed that a total sample of 54 participants and critical F value of 2.79 was required to achieve a power of 0.95.

## Results

Basic CAD patient characteristics are presented in Table 1. The results of the measured 6MWD in CAD patients, classified according to different parameters are presented in Table 2.

**TABLE 1:** Basic characteristics of the patients with coronary artery disease (CAD).

	Males (*N* = 23)		Females (*N* = 44)		Overall (*N* = 67)	
	Mean	SD	Mean	SD	Mean	SD
Age (yrs.)	76.5	7.8	75.1	7.7	75.6	7.7
Body mass (kg)	84.9	12.7	71.2	14.2	75.9	15.1
Body height (cm)	171.5	6.9	158.1	5.1	162.7	8.6
Waist circumference (cm)	104	9	93	11	96.6	11.7
Body mass index (kg/m2)	28.75	3.00	28.34	4.65	28.48	4.14
Waist to height ratio	0.60	0.04	0.59	0.06	0.59	0.06

yrs–years; SD, standard deviation.

**TABLE 2:** Six-minute walk distance in coronary artery disease (CAD) patients.

Parameter		6MWD (meters)	
		Mean	SD
Sex	Men	451	122
	Women	485	69
Test vs. Enright prediction	Measured	473^a^	91
	Predicted	422	57
NYHA classes	Class I	587^b^	31
	Class II	494^c^	28
	Class III	385	100
BMI categories	BMI 18.5–24.9 (normal)	458	96
	BMI 25-0–29.9 (overweight)	471	99
	BMI ≥30 (obese)	487	72
WHtR categories	WHtR <0.5 (no increased risk)	501	20
	WHtR ≥0.5 and <0.6 (increased risk)	497	81
	WHtR ≥0.6 (very high risk)	446	99

NYHA, New York Heart Association; BMI, body mass index; WHtR, Waist to Height Ratio; 6MWD, 6-min walk distance; SD, standard deviation.

a significantly higher measured value; see text for details.

b significantly higher than NYHA II, and NYHA III; see text for details.

c significantly higher than NYHA III; see text for details.

Men reached 451 ± 122 m and women 485 ± 69 m without significant differences between sexes (F = 0.022, *p* = 0.882) when adjusted for age, height, body mass, and WC. Moreover, even in the unadjusted univariate model there were no statistically significant differences between sexes (F = 2.093, *p* = 0.153). Hence, all further regression analyses were performed without differentiating for sex. On the other hand, the covariates WC (F = 5.07, *p* = 0.028) and age (F = 39.46, *p* < 0.001) were significant predictors of 6MWD in this population.

**FIGURE 1:**
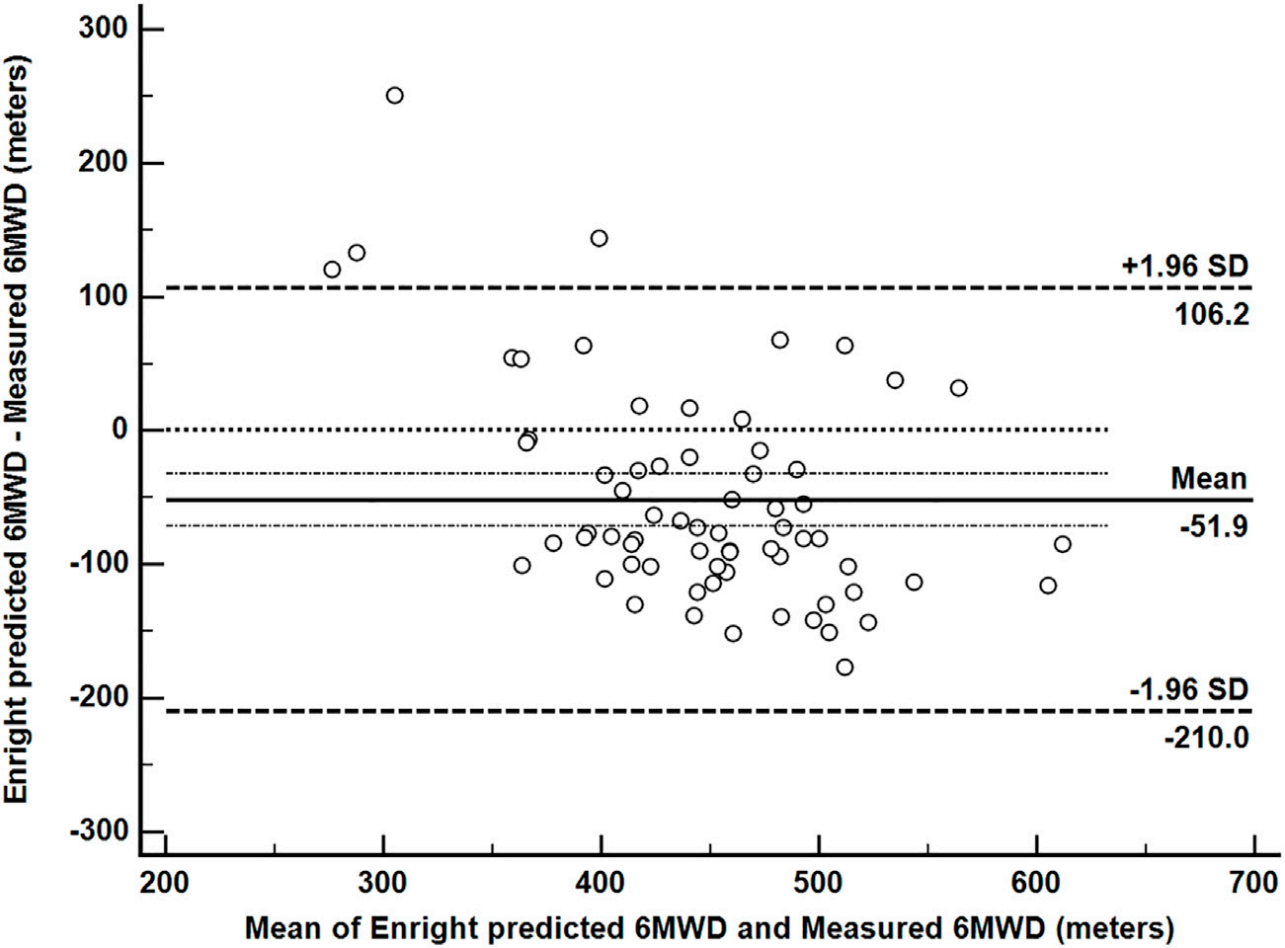
Bland–Altman plot of agreement between the predicted and the measured 6-min walk distance (6MWD). The upper and lower limits of agreement are generally drawn at 1.96 standard deviations (of observed inter-observer differences) above and below the line representing the mean difference (solid line); dotted line is set at zero.

When analyzing the agreement between the predicted and the measured 6MWD using Bland-Altman plot (Figure 1) the prediction equation underestimated the performance in CAD patients for 52 ± 81 m.

This finding was further confirmed when comparing the measured (473 ± 91 m) and the predicted (422 ± 57 m) values of 6MWD in CAD patients using repeated measures ANOVA. We found that the Enright-Sherill prediction equation significantly (F = 27.734, *p* < 0.001) underestimated the 6MWD by 52 ± 81 m. Significantly large effect differences in 6MWD were found for different NYHA classes (F = 14.7, *p* < 0001, effect size = 0.329) where, as expected, patients from NYHA I were performing significantly better than patients from NYHA II (*p* = 0.029) and NYHA III (*p* < 0.001), and NYHA II patients significantly better than NYHA III patients (*p* < 0.001). We found no significant differences in the measured 6MWD among different BMI (*p* = 0.829) or WHtR (*p* = 0.270) categories.

**FIGURE 2:**
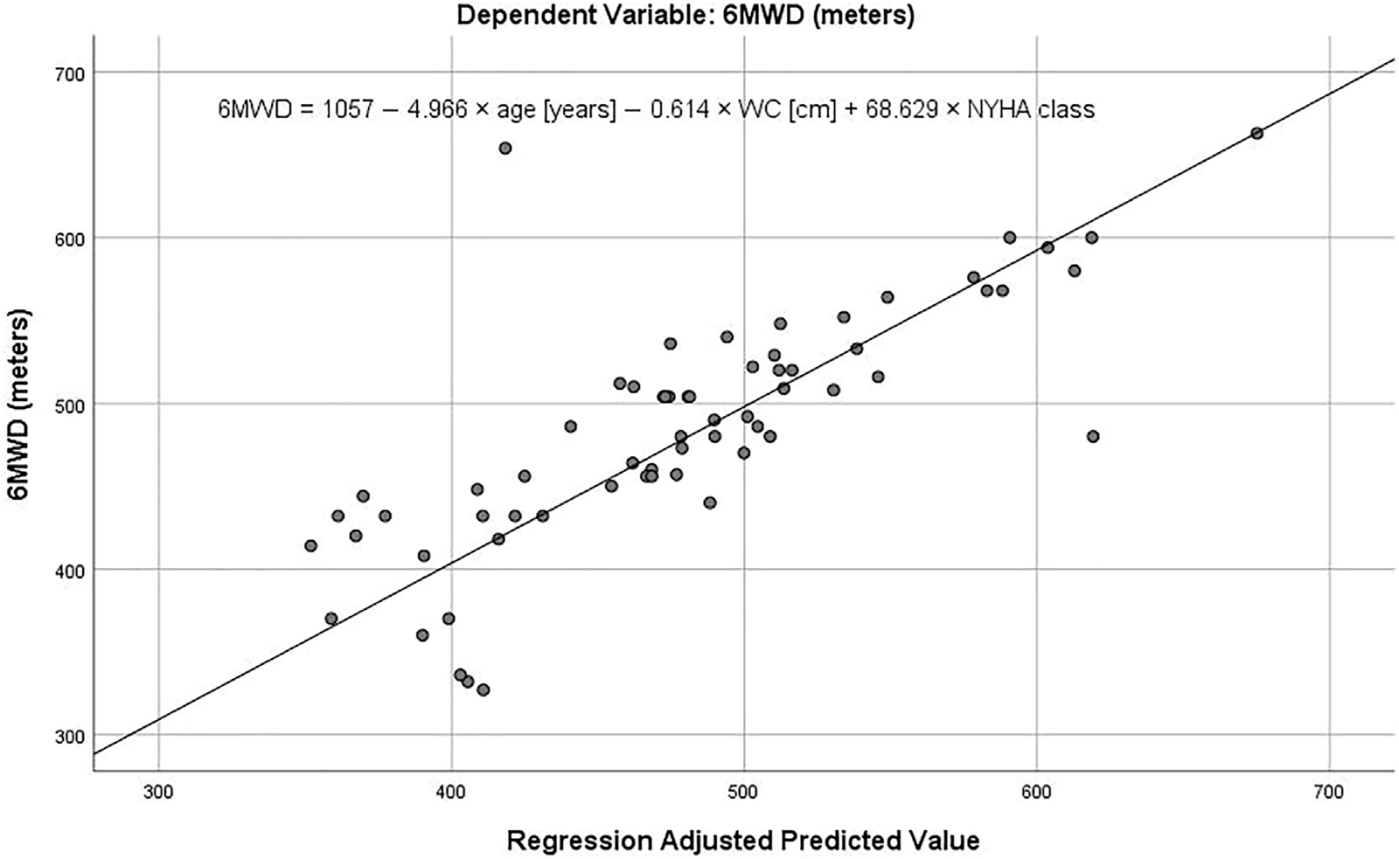
A prediction of the 6-min walk distance (6MWD) in the adjusted multiple linear regression model. WC–waist circumference; NYHA–New York Heart Association. See text for details.

In the single linear regression, only age and NYHA class were significant predictors of 6MWD (Table 3). All multiple regression models including these two predictors were significant, but the highest *R* ^2^ was obtained in the model based on age, WC and NYHA class. Based on this model, a significant regression equation was developed [F (3,63) = 44.663, *p* < 0.001], with a R2 of 0.680. Respectively, our predicted 6MWD in patients equals 1,057 m—4.966 x age (years)—0.614 x WC (cm) - 68.629 x NYHA class, where NYHA class is coded as NYHA I = 1, NYHA II = 2, NYHA III = 3, age is measured in years and WC in centimeters (Figure 2). 6MWD decreased by about 5 m per each year of age, 0.6 m per every centimeter of WC and NYHA I patients walked approximately 69 and 138 m more than NYHA II and NYHA III patients, respectively. Moreover, the regression model considering only age and NYHA class (but not WC) was also significant, with a *R* ^2^ of 0.675.

**TABLE 3:** Single and multiple linear regression model for the prediction of 6MWD in CAD patients.

Constant distance (meters)		Regression Model		*p*-value	Adjusted R2 (model *p*-value)
	Predictors	Coefficient	95% CI		
1,090	Age	−8.162	929.46; 1,250.95	<0.001	0.477 (<0.001)
442	Body mass	0.421	−1.067; 1.909	0.574	0.005 (0.574)
410	Body height	0.389	−2.234; 3.011	0.768	0.001 (0.768)
608	WC	−1.388	−3.281; 0.505	0.148	0.032 (0.148)
695	NYHA class	−102.593	−125.041; −80.144	<0.001	0.562 (<0.001)
1,181	Age	−8.583	−10.76; −6.397	<0.001	0.493 (<0.001)
	Body mass	−0.782	−1.896; 0.332	0.166	
1,302	Age	−8.213	−10.59; −6.10	<0.001	0.489 (<0.001)
	Height	−1.174	−3.11; 0.76	0.230	
1,218	Age	−8.213	−10.33; −6.10	<0.001	0.521 (<0.001)
	Height	0.265	−2.08; 2.61	0.822	
	WC	−1.734	−3.42; −0.05	<0.001	
1,255	Age	−8.162	−10.31; −6.22	<0.001	0.521 (<0.001)
	WC	−1.62	−2.96; −0.28	0.019	
1,057	Age	−4.966	−7.020; −2.911	<0.001	0.680 (<0.001)
	WC	−0.614	−1.778; 0.549	0.295	
	NYHA class	−68.629	−93.086; −44.172	<0.001	
989	Age	−4.738	−6.748; 2.728	<0.001	0.675 (<0.001)
	NYHA class	−72.607	−95.889; −49.325	<0.001	

6MWD, 6-min walk distance; NYHA, New York Heart Association; WC, waist circumference.

## Discussion

The main finding of our study is a new significant regression model explaining 68% of the 6MWD in CAD patients. Accordingly, our prediction equation could be used to evaluate patient performance after conducting a 6MWT. Our results also indicate that: 1) there are no significant differences in 6MWD between men and women with CAD; 2) the Enright-Sherill prediction equation for 6MWD underestimates CAD patient performance on 6MWT; and 3) NYHA class and WC play a significant role in the prediction of 6MWD in CAD patients. To the best of our knowledge, this is the first study to provide such a prediction equation for 6MWD assessment in CAD patients.

Although the Enright- Sherrill prediction equation (Enright and Sherrill, 1998) is adjusted for sex and proposes different calculation of 6MWD for men and women, we found no significant impact of sex on 6MWD in CAD patients. Although studies published in the past 10 years which assessed 6MWT in CAD patients (Table 4) were not analyzing sex related differences, it is worth nothing that they did not report 6MWD separately for men and women (Babu et al., 2010; Worringham et al., 2011; Beatty et al., 2012; Gremeaux et al., 2012; Wu et al., 2013; Lv et al., 2015; Yuniadi et al., 2016; Compostella et al., 2017; Waite et al., 2017; Stewart et al., 2018; Rocco et al., 2019; de Bakker et al., 2020). This finding could indicate that a uniform prediction equation should rather be used in the population of CAD without the need to calculate percentage of prediction differently for men and women. Furthermore, a pooled mean 6MWD that we calculated from previous studies (Table 4) was 454 ± 85 m and was not significantly different from the one that we are reporting [t (2,666) = 1.777, *p* = 0.08], indicating that the mean 6MWD obtained from our sample is within limits of previously reported values. We would like to highlight that in our study, the mean difference between men and women was 34 m, and that although not significant, this difference was above the reported minimal clinically important difference of 25 m (Gremeaux et al., 2011). However, the study by Gremeaux et al. (2011) included 81 patients of which 77 were men, that could partially explain the discrepancy between the two studies.

**TABLE 4:** Six-minute walk distance (6MWD) in patients with coronary heart disease.

Author	Year	*N*	Sex	6MWD (meters)	
				Mean	Std. dev
Current study	2020	65	males/females	473	91
de Bakker	2020	607	males/females	563	77
Stewart	2018	875	males/females	340	117
Rocco	2019	52	males/females	443	62
Waite	2017	11	males/females	237	147
Compostella	2017	139	males	520	114
Gremeaux	2011	81	males/females	488	NA
Yuniadi	2016	26	males/females	352	90
Lv	2015	43	males/females	513	94
Wu	2013	34	males/females	439	87
Beatty	2013	556	males/females	481	36
Gremeaux	2012	30	males/females	490	33
Worringham	2011	134	males/females	524	NA
Babu	2010	15	males/females	470	151

Probably the most interesting finding is the fact that the Enright-Sherill prediction equation, obtained from a healthy population, underestimates the measured performance in CAD patients by 52 m shown by Bland-Altman limits of agreement analysis, meaning that the two procedures (prediction and actual measurement) cannot be used as substitutes for each other. Considering that the reported minimal clinically important difference for 6MWD is 25 m (Gremeaux et al., 2011), we may conclude that the underestimation is both statistically (*p* < 0.001) and clinically significant. When 6MWT is performed in outpatient setting, the results are interpreted to patients as a percentage of the predicted value of 6MWD, which means that in our case we would wrongly classify patient’s CRF for no objective reason. This may decrease patient’s motivation for exercise, as the patient may wrongly percept his performance as clinically acceptable, although he might indeed perform better. Therefore, we conclude that additional properly powered studies are warranted using this proposed, population specific prediction equation to evaluate if it is a more appropriate approach for evaluation and follow-up of CRF in CAD patients. Moreover, the fact that the predicted 6MWD assessed by Enright-Sherill equation underestimated the real 6MWD in patients puts into question the relevance of the equation also in a healthy population and exposes a need for a re-evaluation.

As expected, NYHA class significantly affected the CAD patients’ performance (Table 2). In NYHA 1, where there is no limitation of physical activity and where moderate physical activity does not cause undue fatigue, palpitation and/or dyspnea, the 6MWD was 587 ± 31 m. In NYHA II, where slight limitation of physical activity and comfort at rest exist, the 6MWD was below 500 m (494 ± 28 m), and in NYHA III where there are marked limitations of physical activity with important cardio-respiratory symptoms, 6MWD was below 400 m (385 ± 100 m). In a previous study (Yap et al., 2015), authors did not report any significant differences between NYHA I and II (420 vs. 393 m; *p* = 0.416), but they did report significant differences in mean 6MWD between NYHA II and III (393 vs. 321 m; *p* = 0.014) and III and IV (321 vs. 224 m; *p* = 0.027), respectively. In the best regression prediction model obtained in our study the performance in 6MWT has decreased by 69 m for each change in NYHA class. This has not been shown in any study performed so far. Based on this finding we believe that clinical classification of the heart function performed by cardiologist is crucial to predict and evaluate CRF in this population.

Our calculated regression model that includes age, NYHA class and WC has explained 68% of the variance (with a *R* ^2^ = 0.680) of 6MWD in CAD patients which is much better than the 40% variance explained by the Enright-Sherill equation obtained in a healthy population. Compared to other prediction equations based on a healthy population data (Singh et al., 2014) where *R* ^2^ ranged from 0.09 to 0.77, only three studies reported an equation with R^2^ larger than 0.68 (Poh et al., 2006; Ben Saad et al., 2009; Casanova et al., 2011).

Finally, we would like to stress some strengths and limitations of our study. We have included several important predictors that have not been used previously. We believe that the proposed equation is very feasible given that all predictors are assessed routinely. However, although powered enough the sample size in our study was still relatively small (*N* = 66; sample size calculation was *N* = 54). Nevertheless, seven of 13 similar studies conducted so far (Table 4) included a smaller sample size. Although some could still consider our sample size as a limitation for the accuracy of estimated regression coefficients, studies have also confirmed that even two events per variable can be enough for adequate estimation of regression coefficients, standard errors, and confidence intervals (Austin and Steyerberg, 2015). Another thought worth noting is the fact that in our sample, the number of female participants far outranged the number of male participants, although the prevalence of cardiovascular diseases is usually higher in males. The sex difference in prevalence of CAD diminishes in older age. In addition, it seems that less males than females usually participate in rehabilitation programs, and females are more adherent. This observation urges for additional studies encompassing larger, population representative samples and stresses the need for a more efficient promotion of importance of cardiovascular rehabilitation, especially targeting male population.

In conclusion, we have provided a new prediction equation based on multivariate regression model for 6 MWD estimation, considering the variables age, NYHA class and WC as predictors. To the best of our knowledge, this is the first study to challenge prediction of 6MWD using this approach. The prediction equation developed in this study may represent a good substitute for currently used predictions from healthy population in order to avoid the possibility of underestimation or overestimation of patient performance. However, it should be stressed that our results are not providing evidence for this, as analysis of proposed equation in an independent CAD patients sample is necessary for validation and potential clinical use. Nevertheless, the results of our study stress the importance of regular and actual walking ability testing in patients with stable CAD to obtain their CRF, rather than simply predicting it from regression equations obtained from non-representative or non-comparable samples. Actual testing and better data interpretation using more objective prediction equation enables a more realistic and obtainable exercise goal setting in CAD patients.

## Data Availability

The original contributions presented in the study are included in the article/Supplementary Material, further inquiries can be directed to the corresponding author.
